# Bis(μ-2-methyl­quinolin-8-olato)-κ^3^ *N*,*O*:*O*;κ^3^ *O*:*N*,*O*-bis­[(acetato-κ^2^ *O*,*O*′)lead(II)]

**DOI:** 10.1107/S1600536809003560

**Published:** 2009-02-11

**Authors:** Gholamhossein Mohammadnezhad Sh., Mostafa M. Amini, Seik Weng Ng

**Affiliations:** a Department of Chemistry, General Campus, Shahid Beheshti University, Tehran 1983963113, Iran; b Department of Chemistry, University of Malaya, 50603 Kuala Lumpur, Malaysia

## Abstract

Both independent Pb^II^ atoms in the title compound, [Pb_2_(C_10_H_8_NO)_2_(C_2_H_3_O_2_)_2_], are chelated by acetate and substituted quinolin-8-olate anions; the O atoms of the quinolin-8-olates also bridge to confer a five-coordinate status to each metal center. The geometry approximates a distorted Ψ-*fac* octa­hedron in which one of the sites is occupied by a stereochemically active lone pair.

## Related literature

The structural chemistry of lead(II) 8-hydroxy­quinolinates has been reviewed, including bis­(*μ*-acetato)diacetatotetra­kis(*μ*-quinolin-8-olato)tetra­lead dihydrate (Shahverdizadeh *et al.*, 2008 ▶).
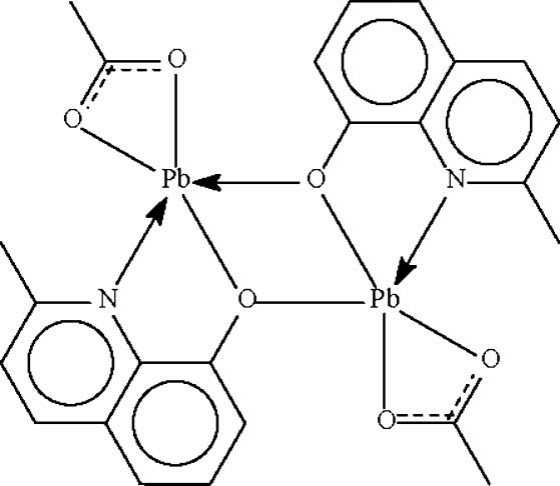


## Experimental

### 

#### Crystal data

[Pb_2_(C_10_H_8_NO)_2_(C_2_H_3_O_2_)_2_]
*M* *_r_* = 848.82
Orthorhombic, 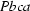

*a* = 13.7421 (2) Å
*b* = 18.0682 (3) Å
*c* = 18.6113 (4) Å
*V* = 4621.1 (1) Å^3^
*Z* = 8
Mo *K*α radiation
μ = 14.60 mm^−1^
*T* = 100 (2) K
0.20 × 0.10 × 0.08 mm

#### Data collection

Bruker SMART APEX diffractometer
Absorption correction: multi-scan (*SADABS*; Sheldrick, 1996 ▶) *T*_min_ = 0.158, *T*_max_ = 0.388 (expected range = 0.127–0.311)
33016 measured reflections
4063 independent reflections
3133 reflections with *I* > 2σ(*I*)
*R*_int_ = 0.090

#### Refinement

*R*[*F* ^2^ > 2σ(*F* ^2^)] = 0.046
*wR*(*F* ^2^) = 0.115
*S* = 1.45
4063 reflections
264 parameters
192 restraints
H-atom parameters constrained
Δρ_max_ = 4.37 e Å^−3^
Δρ_min_ = −2.70 e Å^−3^

### 

Data collection: *APEX2* (Bruker, 2008 ▶); cell refinement: *SAINT* (Bruker, 2008 ▶); data reduction: *SAINT*; program(s) used to solve structure: *SHELXS97* (Sheldrick, 2008 ▶); program(s) used to refine structure: *SHELXL97* (Sheldrick, 2008 ▶); molecular graphics: *X-SEED* (Barbour, 2001 ▶); software used to prepare material for publication: *publCIF* (Westrip, 2009 ▶).

## Supplementary Material

Crystal structure: contains datablocks global, I. DOI: 10.1107/S1600536809003560/tk2364sup1.cif

Structure factors: contains datablocks I. DOI: 10.1107/S1600536809003560/tk2364Isup2.hkl

Additional supplementary materials:  crystallographic information; 3D view; checkCIF report

## Figures and Tables

**Fig. 1.**
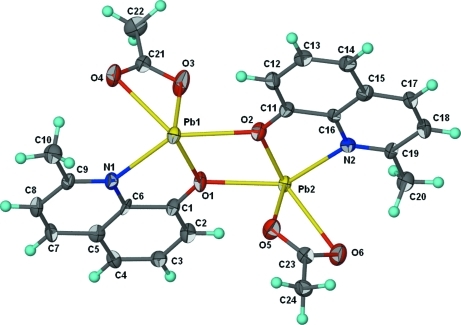
Thermal ellipsoid plot (Barbour, 2001) of Pb2(C2H3O2)2(C10H8NO)2 at the 70% probability level. Hydrogen atoms are drawn as spheres of arbitrary radius.

## supplementary crystallographic information

### Experimental

Lead acetate (0.38 g, 1 mmol) and 2-methyl-8-hydroxyquinoline (0.32 g, 2 mmol) were loaded into a convection tube; the tube was filled with dry methanol and kept at 333 K. Crystals were collected from the side arm after 1 day.

### Refinement

Carbon-bound H-atoms were placed in calculated positions (C—H 0.93 to 0.98 Å) and were included in the refinement in the riding model approximation, with *U*(H) set to 1.2 to 1.5*U*(C).

The quinolinyl ring was refined as a rigid group with C-C = 1.39 Å. The crystal diffracted strongly owing to two extremely heavy metal atoms. However, their presence introduced severe absorption problems that could not be corrected analytically as the crystal did not have regular faces. Although a sphere of reflections was measured, multi-scan treatment only marginally improved the quality. The final difference Fourier map had large peaks/deep holes near the lead atoms. The anisotropic displacement factors of the carbon, nitrogen and oxygen atoms had to be restrained to be nearly isotropic.

### Crystal data

**Table:**

[Pb_2_(C_10_H_8_NO)_2_(C_2_H_3_O_2_)_2_]	*F*(000) = 3136
*M**_r_* = 848.82	*D*_x_ = 2.440 Mg m^−^^3^
Orthorhombic, *P**b**c**a*	Mo *K*α radiation, λ = 0.71073 Å
Hall symbol: -P 2ac 2ab	Cell parameters from 6225 reflections
*a* = 13.7421 (2) Å	θ = 2.2–28.3°
*b* = 18.0682 (3) Å	µ = 14.60 mm^−^^1^
*c* = 18.6113 (4) Å	*T* = 100 K
*V* = 4621.1 (1) Å^3^	Block, yellow
*Z* = 8	0.20 × 0.10 × 0.08 mm

### Data collection

**Table:**

Bruker SMART APEX diffractometer	4063 independent reflections
Radiation source: fine-focus sealed tube	3133 reflections with *I* > 2σ(*I*)
graphite	*R*_int_ = 0.090
ω scans	θ_max_ = 25.0°, θ_min_ = 2.2°
Absorption correction: multi-scan (*SADABS*; Sheldrick, 1996)	*h* = −16→16
*T*_min_ = 0.158, *T*_max_ = 0.388	*k* = −21→21
33016 measured reflections	*l* = −22→21

### Refinement

**Table:**

Refinement on *F*^2^	Primary atom site location: structure-invariant direct methods
Least-squares matrix: full	Secondary atom site location: difference Fourier map
*R*[*F*^2^ > 2σ(*F*^2^)] = 0.046	Hydrogen site location: inferred from neighbouring sites
*wR*(*F*^2^) = 0.115	H-atom parameters constrained
*S* = 1.45	*w* = 1/[σ^2^(*F*_o_^2^) + (0.0388*P*)^2^ + 1*P*] where *P* = (*F*_o_^2^ + 2*F*_c_^2^)/3
4063 reflections	(Δ/σ)_max_ = 0.001
264 parameters	Δρ_max_ = 4.37 e Å^−^^3^
192 restraints	Δρ_min_ = −2.70 e Å^−^^3^

### Fractional atomic coordinates and isotropic or equivalent isotropic displacement parameters (Å^2^)

**Table:**

	*x*	*y*	*z*	*U*_iso_*/*U*_eq_	
Pb1	0.28159 (3)	0.37522 (2)	0.52835 (2)	0.01649 (15)	
Pb2	0.56385 (3)	0.38486 (2)	0.48211 (2)	0.01580 (14)	
O1	0.3933 (6)	0.4289 (4)	0.4554 (4)	0.0190 (18)	
O2	0.4542 (5)	0.3401 (4)	0.5634 (4)	0.0193 (17)	
O3	0.3217 (6)	0.4747 (4)	0.6014 (5)	0.029 (2)	
O4	0.1631 (6)	0.4725 (4)	0.5938 (5)	0.027 (2)	
O5	0.5071 (6)	0.2851 (4)	0.4186 (4)	0.027 (2)	
O6	0.6648 (6)	0.2738 (4)	0.3994 (5)	0.030 (2)	
C1	0.3673 (3)	0.4758 (3)	0.4007 (3)	0.017 (2)	
C2	0.4354 (3)	0.5134 (3)	0.3593 (3)	0.021 (3)	
H2	0.5029	0.5076	0.3689	0.026*	
C3	0.4048 (3)	0.5595 (3)	0.3040 (3)	0.020 (3)	
H3	0.4514	0.5852	0.2757	0.024*	
C4	0.3061 (4)	0.5680 (3)	0.2900 (3)	0.019 (3)	
H4	0.2852	0.5996	0.2522	0.023*	
C5	0.2380 (3)	0.53044 (19)	0.3314 (2)	0.021 (3)	
C6	0.2686 (3)	0.48434 (18)	0.3868 (2)	0.015 (2)	
N1	0.2004 (3)	0.4467 (2)	0.4282 (2)	0.016 (2)	
C9	0.1017 (3)	0.4552 (3)	0.4142 (3)	0.017 (2)	
C8	0.0711 (3)	0.5013 (3)	0.3589 (3)	0.022 (3)	
H8	0.0036	0.5072	0.3494	0.026*	
C7	0.1392 (3)	0.5389 (3)	0.3175 (3)	0.019 (3)	
H7	0.1183	0.5705	0.2797	0.023*	
C10	0.0363 (9)	0.4109 (6)	0.4591 (7)	0.025 (3)	
H10A	0.0535	0.3585	0.4547	0.038*	
H10B	−0.0310	0.4183	0.4432	0.038*	
H10C	0.0427	0.4264	0.5093	0.038*	
C11	0.4812 (3)	0.2885 (3)	0.6133 (3)	0.017 (2)	
C12	0.4148 (3)	0.2545 (3)	0.6589 (3)	0.024 (3)	
H12	0.3475	0.2659	0.6554	0.028*	
C13	0.4469 (3)	0.2036 (3)	0.7097 (3)	0.018 (3)	
H13	0.4016	0.1803	0.7409	0.022*	
C14	0.5454 (4)	0.1868 (3)	0.7148 (3)	0.022 (3)	
H14	0.5673	0.1521	0.7496	0.026*	
C15	0.6118 (3)	0.22089 (19)	0.6692 (2)	0.018 (3)	
C16	0.5797 (3)	0.27173 (19)	0.6184 (2)	0.013 (2)	
N2	0.6460 (3)	0.3058 (2)	0.5728 (2)	0.015 (2)	
C19	0.7445 (3)	0.2890 (3)	0.5779 (3)	0.017 (3)	
C18	0.7766 (3)	0.2382 (3)	0.6287 (3)	0.021 (3)	
H18	0.8439	0.2267	0.6322	0.026*	
C17	0.7102 (3)	0.2041 (3)	0.6743 (3)	0.017 (2)	
H17	0.7322	0.1694	0.7090	0.021*	
C20	0.8077 (9)	0.3291 (6)	0.5282 (7)	0.024 (3)	
H20A	0.8022	0.3824	0.5372	0.036*	
H20B	0.7880	0.3184	0.4787	0.036*	
H20C	0.8752	0.3134	0.5355	0.036*	
C21	0.2377 (9)	0.5005 (6)	0.6175 (6)	0.019 (3)	
C22	0.2349 (10)	0.5681 (6)	0.6657 (7)	0.031 (3)	
H22A	0.2560	0.6116	0.6385	0.047*	
H22B	0.2785	0.5605	0.7067	0.047*	
H22C	0.1683	0.5757	0.6831	0.047*	
C23	0.5803 (8)	0.2518 (6)	0.3911 (6)	0.017 (3)	
C24	0.5582 (9)	0.1846 (6)	0.3472 (6)	0.023 (3)	
H24A	0.5722	0.1948	0.2965	0.035*	
H24B	0.4894	0.1717	0.3526	0.035*	
H24C	0.5987	0.1432	0.3637	0.035*	

### Atomic displacement parameters (Å^2^)

**Table:**

	*U*^11^	*U*^22^	*U*^33^	*U*^12^	*U*^13^	*U*^23^
Pb1	0.0121 (3)	0.0160 (2)	0.0214 (3)	−0.00051 (15)	0.00086 (17)	0.00142 (16)
Pb2	0.0108 (2)	0.0164 (2)	0.0201 (3)	0.00012 (15)	0.00039 (17)	0.00064 (17)
O1	0.011 (4)	0.024 (4)	0.023 (4)	0.004 (3)	0.002 (3)	0.008 (3)
O2	0.013 (4)	0.021 (4)	0.023 (4)	0.003 (3)	0.002 (3)	0.004 (3)
O3	0.013 (5)	0.027 (4)	0.047 (5)	−0.002 (3)	0.000 (4)	−0.013 (4)
O4	0.018 (5)	0.031 (4)	0.033 (5)	0.003 (3)	−0.001 (4)	−0.008 (3)
O5	0.018 (4)	0.027 (4)	0.034 (5)	−0.005 (3)	−0.001 (4)	−0.012 (3)
O6	0.020 (5)	0.034 (4)	0.036 (5)	0.001 (4)	0.000 (4)	−0.004 (4)
C1	0.013 (6)	0.018 (5)	0.019 (6)	−0.004 (4)	0.004 (4)	−0.001 (4)
C2	0.018 (6)	0.018 (5)	0.028 (6)	−0.003 (4)	0.001 (5)	0.002 (4)
C3	0.018 (6)	0.022 (5)	0.020 (6)	−0.005 (4)	0.002 (5)	−0.001 (4)
C4	0.020 (6)	0.018 (5)	0.020 (6)	−0.002 (4)	−0.006 (5)	−0.001 (4)
C5	0.022 (6)	0.014 (5)	0.025 (6)	0.003 (4)	0.000 (5)	−0.002 (4)
C6	0.006 (5)	0.018 (5)	0.020 (6)	0.003 (4)	0.005 (4)	−0.002 (4)
N1	0.018 (5)	0.012 (4)	0.018 (5)	0.010 (3)	−0.003 (4)	0.002 (3)
C9	0.011 (5)	0.017 (5)	0.024 (6)	0.001 (4)	0.001 (4)	−0.007 (4)
C8	0.014 (6)	0.024 (5)	0.027 (6)	0.003 (4)	−0.005 (5)	0.001 (4)
C7	0.016 (6)	0.018 (5)	0.023 (6)	−0.001 (4)	−0.004 (5)	0.004 (4)
C10	0.020 (6)	0.027 (5)	0.029 (6)	−0.002 (5)	0.000 (5)	−0.002 (5)
C11	0.014 (6)	0.024 (5)	0.014 (5)	0.003 (4)	0.001 (4)	0.000 (4)
C12	0.020 (6)	0.027 (5)	0.025 (6)	−0.001 (4)	0.002 (5)	−0.002 (5)
C13	0.016 (6)	0.019 (5)	0.020 (6)	−0.007 (4)	0.003 (5)	0.000 (4)
C14	0.026 (6)	0.023 (5)	0.016 (6)	−0.004 (5)	0.002 (5)	0.001 (4)
C15	0.020 (6)	0.014 (5)	0.021 (6)	0.002 (4)	−0.001 (5)	−0.005 (4)
C16	0.012 (6)	0.016 (5)	0.012 (5)	−0.003 (4)	−0.001 (4)	−0.005 (4)
N2	0.020 (5)	0.013 (4)	0.013 (5)	0.005 (4)	−0.001 (4)	0.001 (3)
C19	0.013 (6)	0.019 (5)	0.018 (6)	−0.001 (4)	0.002 (4)	0.000 (4)
C18	0.016 (6)	0.025 (5)	0.023 (6)	0.005 (4)	−0.002 (5)	0.000 (4)
C17	0.017 (6)	0.016 (5)	0.019 (6)	0.003 (4)	−0.002 (5)	0.004 (4)
C20	0.020 (6)	0.021 (5)	0.031 (6)	0.002 (4)	−0.003 (5)	−0.001 (4)
C21	0.017 (6)	0.017 (5)	0.022 (6)	−0.002 (4)	−0.001 (5)	0.006 (4)
C22	0.036 (7)	0.022 (5)	0.036 (7)	−0.002 (5)	0.005 (6)	−0.003 (5)
C23	0.015 (6)	0.018 (5)	0.019 (5)	−0.003 (4)	0.000 (5)	0.003 (4)
C24	0.023 (6)	0.023 (5)	0.023 (6)	−0.001 (5)	−0.001 (5)	0.000 (4)

### Geometric parameters (Å, °)

**Table:**

Pb1—O1	2.267 (8)	C7—H7	0.9500
Pb1—O3	2.321 (8)	C10—H10A	0.9800
Pb1—N1	2.527 (4)	C10—H10B	0.9800
Pb1—O2	2.541 (7)	C10—H10C	0.9800
Pb1—O4	2.688 (8)	C11—C12	1.3900
Pb2—O2	2.283 (7)	C11—C16	1.3900
Pb2—O5	2.292 (7)	C12—C13	1.3900
Pb2—N2	2.482 (4)	C12—H12	0.9500
Pb2—O1	2.525 (7)	C13—C14	1.3900
Pb2—O4	6.095 (8)	C13—H13	0.9500
O1—C1	1.372 (8)	C14—C15	1.3900
O2—C11	1.366 (8)	C14—H14	0.9500
O3—C21	1.280 (14)	C15—C16	1.3900
O4—C21	1.226 (14)	C15—C17	1.3900
O5—C23	1.279 (13)	C16—N2	1.3900
O6—C23	1.237 (13)	N2—C19	1.3900
C1—C2	1.3900	C19—C18	1.3900
C1—C6	1.3900	C19—C20	1.460 (12)
C2—C3	1.3900	C18—C17	1.3900
C2—H2	0.9500	C18—H18	0.9500
C3—C4	1.3900	C17—H17	0.9500
C3—H3	0.9500	C20—H20A	0.9800
C4—C5	1.3900	C20—H20B	0.9800
C4—H4	0.9500	C20—H20C	0.9800
C5—C6	1.3900	C21—C22	1.515 (16)
C5—C7	1.3900	C22—H22A	0.9800
C6—N1	1.3900	C22—H22B	0.9800
N1—C9	1.3900	C22—H22C	0.9800
C9—C8	1.3900	C23—C24	1.494 (15)
C9—C10	1.465 (12)	C24—H24A	0.9800
C8—C7	1.3900	C24—H24B	0.9800
C8—H8	0.9500	C24—H24C	0.9800
			
O1—Pb1—O3	81.9 (3)	C9—C10—H10A	109.5
O1—Pb1—N1	68.8 (2)	C9—C10—H10B	109.5
O3—Pb1—N1	98.1 (2)	H10A—C10—H10B	109.5
O1—Pb1—O2	68.2 (2)	C9—C10—H10C	109.5
O3—Pb1—O2	79.7 (3)	H10A—C10—H10C	109.5
N1—Pb1—O2	136.80 (19)	H10B—C10—H10C	109.5
O1—Pb1—O4	113.7 (2)	O2—C11—C12	122.6 (4)
O3—Pb1—O4	51.1 (3)	O2—C11—C16	117.4 (4)
N1—Pb1—O4	74.5 (2)	C12—C11—C16	120.0
O2—Pb1—O4	127.8 (2)	C11—C12—C13	120.0
O2—Pb2—O5	80.7 (3)	C11—C12—H12	120.0
O2—Pb2—N2	69.3 (2)	C13—C12—H12	120.0
O5—Pb2—N2	93.0 (2)	C14—C13—C12	120.0
O2—Pb2—O1	68.3 (2)	C14—C13—H13	120.0
O5—Pb2—O1	80.3 (3)	C12—C13—H13	120.0
N2—Pb2—O1	137.5 (2)	C13—C14—C15	120.0
O2—Pb2—O4	43.12 (19)	C13—C14—H14	120.0
O5—Pb2—O4	94.2 (2)	C15—C14—H14	120.0
N2—Pb2—O4	109.19 (13)	C14—C15—C16	120.0
O1—Pb2—O4	31.4 (2)	C14—C15—C17	120.0
C1—O1—Pb1	122.2 (5)	C16—C15—C17	120.0
C1—O1—Pb2	125.6 (5)	N2—C16—C15	120.0
Pb1—O1—Pb2	112.1 (3)	N2—C16—C11	120.0
C11—O2—Pb2	120.9 (5)	C15—C16—C11	120.0
C11—O2—Pb1	126.7 (5)	C16—N2—C19	120.0
Pb2—O2—Pb1	111.0 (3)	C16—N2—Pb2	111.83 (17)
C21—O3—Pb1	101.8 (7)	C19—N2—Pb2	127.96 (17)
C21—O4—Pb1	85.8 (7)	N2—C19—C18	120.0
C21—O4—Pb2	58.3 (7)	N2—C19—C20	115.3 (6)
C23—O5—Pb2	108.0 (7)	C18—C19—C20	124.7 (6)
O1—C1—C2	122.5 (4)	C17—C18—C19	120.0
O1—C1—C6	117.5 (4)	C17—C18—H18	120.0
C2—C1—C6	120.0	C19—C18—H18	120.0
C3—C2—C1	120.0	C18—C17—C15	120.0
C3—C2—H2	120.0	C18—C17—H17	120.0
C1—C2—H2	120.0	C15—C17—H17	120.0
C2—C3—C4	120.0	C19—C20—H20A	109.5
C2—C3—H3	120.0	C19—C20—H20B	109.5
C4—C3—H3	120.0	H20A—C20—H20B	109.5
C5—C4—C3	120.0	C19—C20—H20C	109.5
C5—C4—H4	120.0	H20A—C20—H20C	109.5
C3—C4—H4	120.0	H20B—C20—H20C	109.5
C6—C5—C4	120.0	O4—C21—O3	121.3 (10)
C6—C5—C7	120.0	O4—C21—C22	121.6 (11)
C4—C5—C7	120.0	O3—C21—C22	117.1 (11)
N1—C6—C5	120.0	C21—C22—H22A	109.5
N1—C6—C1	120.0	C21—C22—H22B	109.5
C5—C6—C1	120.0	H22A—C22—H22B	109.5
C6—N1—C9	120.0	C21—C22—H22C	109.5
C6—N1—Pb1	111.19 (17)	H22A—C22—H22C	109.5
C9—N1—Pb1	128.68 (17)	H22B—C22—H22C	109.5
C8—C9—N1	120.0	O6—C23—O5	122.4 (10)
C8—C9—C10	124.4 (6)	O6—C23—C24	121.3 (11)
N1—C9—C10	115.6 (6)	O5—C23—C24	116.2 (10)
C9—C8—C7	120.0	C23—C24—H24A	109.5
C9—C8—H8	120.0	C23—C24—H24B	109.5
C7—C8—H8	120.0	H24A—C24—H24B	109.5
C8—C7—C5	120.0	C23—C24—H24C	109.5
C8—C7—H7	120.0	H24A—C24—H24C	109.5
C5—C7—H7	120.0	H24B—C24—H24C	109.5
			
O3—Pb1—O1—C1	−96.4 (6)	C2—C1—C6—N1	180.0
N1—Pb1—O1—C1	5.6 (5)	O1—C1—C6—C5	−179.2 (6)
O2—Pb1—O1—C1	−178.6 (7)	C2—C1—C6—C5	0.0
O4—Pb1—O1—C1	−55.5 (7)	C5—C6—N1—C9	0.0
O3—Pb1—O1—Pb2	87.6 (3)	C1—C6—N1—C9	180.0
N1—Pb1—O1—Pb2	−170.4 (4)	C5—C6—N1—Pb1	−176.3 (3)
O2—Pb1—O1—Pb2	5.5 (3)	C1—C6—N1—Pb1	3.7 (3)
O4—Pb1—O1—Pb2	128.5 (3)	O1—Pb1—N1—C6	−4.6 (2)
O2—Pb2—O1—C1	178.1 (7)	O3—Pb1—N1—C6	73.3 (3)
O5—Pb2—O1—C1	−98.1 (6)	O2—Pb1—N1—C6	−10.3 (4)
N2—Pb2—O1—C1	178.1 (5)	O4—Pb1—N1—C6	119.1 (3)
O4—Pb2—O1—C1	146.9 (8)	O1—Pb1—N1—C9	179.5 (3)
O2—Pb2—O1—Pb1	−6.1 (3)	O3—Pb1—N1—C9	−102.5 (3)
O5—Pb2—O1—Pb1	77.7 (3)	O2—Pb1—N1—C9	173.9 (3)
N2—Pb2—O1—Pb1	−6.1 (5)	O4—Pb1—N1—C9	−56.8 (3)
O4—Pb2—O1—Pb1	−37.3 (2)	C6—N1—C9—C8	0.0
O5—Pb2—O2—C11	89.3 (6)	Pb1—N1—C9—C8	175.5 (3)
N2—Pb2—O2—C11	−7.5 (5)	C6—N1—C9—C10	177.7 (6)
O1—Pb2—O2—C11	172.5 (6)	Pb1—N1—C9—C10	−6.8 (6)
O4—Pb2—O2—C11	−164.3 (7)	N1—C9—C8—C7	0.0
O5—Pb2—O2—Pb1	−77.7 (3)	C10—C9—C8—C7	−177.4 (7)
N2—Pb2—O2—Pb1	−174.6 (3)	C9—C8—C7—C5	0.0
O1—Pb2—O2—Pb1	5.4 (2)	C6—C5—C7—C8	0.0
O4—Pb2—O2—Pb1	28.68 (16)	C4—C5—C7—C8	180.0
O1—Pb1—O2—C11	−172.1 (7)	Pb2—O2—C11—C12	−173.4 (2)
O3—Pb1—O2—C11	102.6 (6)	Pb1—O2—C11—C12	−8.6 (7)
N1—Pb1—O2—C11	−166.5 (5)	Pb2—O2—C11—C16	8.0 (7)
O4—Pb1—O2—C11	84.2 (6)	Pb1—O2—C11—C16	172.8 (3)
O1—Pb1—O2—Pb2	−6.0 (3)	O2—C11—C12—C13	−178.6 (6)
O3—Pb1—O2—Pb2	−91.3 (3)	C16—C11—C12—C13	0.0
N1—Pb1—O2—Pb2	−0.4 (5)	C11—C12—C13—C14	0.0
O4—Pb1—O2—Pb2	−109.7 (3)	C12—C13—C14—C15	0.0
O1—Pb1—O3—C21	130.1 (7)	C13—C14—C15—C16	0.0
N1—Pb1—O3—C21	63.0 (7)	C13—C14—C15—C17	180.0
O2—Pb1—O3—C21	−160.8 (7)	C14—C15—C16—N2	180.0
O4—Pb1—O3—C21	0.5 (6)	C17—C15—C16—N2	0.0
O1—Pb1—O4—C21	−57.0 (7)	C14—C15—C16—C11	0.0
O3—Pb1—O4—C21	−0.5 (7)	C17—C15—C16—C11	180.0
N1—Pb1—O4—C21	−114.9 (7)	O2—C11—C16—N2	−1.4 (6)
O2—Pb1—O4—C21	23.0 (8)	C12—C11—C16—N2	180.0
O1—Pb1—O4—Pb2	−37.8 (2)	O2—C11—C16—C15	178.6 (6)
O3—Pb1—O4—Pb2	18.6 (4)	C12—C11—C16—C15	0.0
N1—Pb1—O4—Pb2	−95.7 (2)	C15—C16—N2—C19	0.0
O2—Pb1—O4—Pb2	42.2 (2)	C11—C16—N2—C19	180.0
O2—Pb2—O4—C21	97.5 (8)	C15—C16—N2—Pb2	175.1 (3)
O5—Pb2—O4—C21	169.2 (8)	C11—C16—N2—Pb2	−4.9 (3)
N2—Pb2—O4—C21	74.5 (8)	O2—Pb2—N2—C16	6.2 (2)
O1—Pb2—O4—C21	−127.3 (8)	O5—Pb2—N2—C16	−72.7 (3)
O2—Pb2—O4—Pb1	−59.9 (3)	O1—Pb2—N2—C16	6.2 (4)
O5—Pb2—O4—Pb1	11.8 (3)	O4—Pb2—N2—C16	22.8 (2)
N2—Pb2—O4—Pb1	−82.9 (3)	O2—Pb2—N2—C19	−179.2 (3)
O1—Pb2—O4—Pb1	75.4 (4)	O5—Pb2—N2—C19	101.9 (3)
O2—Pb2—O5—C23	−135.3 (7)	O1—Pb2—N2—C19	−179.2 (3)
N2—Pb2—O5—C23	−66.8 (7)	O4—Pb2—N2—C19	−162.6 (2)
O1—Pb2—O5—C23	155.4 (7)	C16—N2—C19—C18	0.0
O4—Pb2—O5—C23	−176.4 (7)	Pb2—N2—C19—C18	−174.2 (3)
Pb1—O1—C1—C2	174.9 (3)	C16—N2—C19—C20	−178.9 (6)
Pb2—O1—C1—C2	−9.7 (7)	Pb2—N2—C19—C20	6.9 (6)
Pb1—O1—C1—C6	−5.9 (7)	N2—C19—C18—C17	0.0
Pb2—O1—C1—C6	169.5 (3)	C20—C19—C18—C17	178.8 (7)
O1—C1—C2—C3	179.2 (6)	C19—C18—C17—C15	0.0
C6—C1—C2—C3	0.0	C14—C15—C17—C18	180.0
C1—C2—C3—C4	0.0	C16—C15—C17—C18	0.0
C2—C3—C4—C5	0.0	Pb1—O4—C21—O3	0.9 (11)
C3—C4—C5—C6	0.0	Pb2—O4—C21—O3	−10.0 (8)
C3—C4—C5—C7	180.0	Pb1—O4—C21—C22	179.5 (10)
C4—C5—C6—N1	180.0	Pb2—O4—C21—C22	168.6 (13)
C7—C5—C6—N1	0.0	Pb1—O3—C21—O4	−1.0 (13)
C4—C5—C6—C1	0.0	Pb1—O3—C21—C22	−179.7 (8)
C7—C5—C6—C1	180.0	Pb2—O5—C23—O6	−0.7 (13)
O1—C1—C6—N1	0.8 (5)	Pb2—O5—C23—C24	180.0 (7)
